# Reading comprehension improvement in autism

**DOI:** 10.3389/fpsyt.2024.1292018

**Published:** 2024-03-18

**Authors:** Meagan Beckerson, Courtney Paisley, Donna Murdaugh, Haley Holm, Amy Lemelman, Alyssa Spencer, Sarah O’Kelley, Rajesh Kana

**Affiliations:** ^1^ Department of Psychology, College of Arts and Sciences, University of Alabama at Birmingham, Birmingham, AL, United States; ^2^ Department of Developmental Pediatrics, Children’s Hospital Colorado, Aurora, CO, United States; ^3^ Department of Pediatrics, University of Alabama Birmingham (UAB), Birmingham, AL, United States; ^4^ Department of Neuropsychology, Children’s Healthcare of Atlanta, Atlanta, GA, United States; ^5^ Department of Psychiatry, Weill Cornell Medicine, Cornell University, New York, NY, United States; ^6^ Department of Psychology, University of Alabama, Tuscaloosa, AL, United States

**Keywords:** autism, reading comprehension, visualizing/verbalizing, intervention, verbal memory

## Abstract

**Introduction:**

A subset of autistic children excel at word decoding but have difficulty with reading comprehension (i.e., the *discrepant poor comprehender reading profile*). Prior research suggests the Visualizing and Verbalizing (V/V) for language comprehension and thinking intervention helps improve reading comprehension in autistic children with this reading profile. Previous studies have demonstrated the role of vocabulary, memory, and social functioning in reading comprehension; however, predictors and moderators of reading comprehension within this specific profile of autistic readers have not been thoroughly explored.

**Methods:**

In this study, we examined the effectiveness of the V/V intervention by comparing reading comprehension scores between groups and across time. Participants included a sample of autistic children (AUT-EXP; n=22) and a waitlist control group of autistic children (AUT-WLC; n=17) with reading comprehension difficulties, as well as a sample of non-autistic children (Non-AUT; n=26) (all age 8-13 years). AUT-EXP and AUT-WLC groups completed a battery of cognitive assessments during pre and post tests. We also analyzed whether cognitive assessment scores predicted reading comprehension, and examined the moderating effects of group (AUT-EXP vs. AUT-WLC) on these relationships.

**Results:**

The AUT-EXP group significantly improved in their pre to post reading comprehension scores (*t*(21)=4.19, *p*<.001, *d*=.89), whereas the AUT-WLC group did not. Verbal memory significantly predicted reading comprehension, though group did not moderate relationships between cognitive test performance and reading comprehension.

**Discussion:**

Results suggest that the V/V intervention may help improve reading comprehension for autistic children with the discrepant poor comprehender reading profile. Additionally, strategies for improving verbal memory may indirectly enhance reading comprehension in autistic children with this reading profile.

## 1 Introduction

Autism Spectrum Disorder entails a broad neurodevelopmental condition that is diagnosed based on differences in social communication, and the presence of restricted and repetitive patterns of behaviors and interests (1). Recent estimates report a rise in the prevalence of autism, as 1 in 44 children in the United States are estimated to meet diagnostic criteria (2). Autistic individuals vary considerably in their strengths, weaknesses, and the extent of support needed, highlighting the widespread heterogeneity of this complex condition. As such, previous studies have shown that academic achievement varies widely among autistic children, ranging from exceptional performance to severely impaired (3, 4). A previous study using the academic achievement discrepancy model showed that 60% of autistic children obtained a significantly lower achievement level than they were predicted to achieve in spelling, word reading, or basic number skills (5). Difficulty with social interaction, language, and communication, which are common in autism, can lead to difficulties with reading comprehension (6), making inferences from conversations or written stories (7), and in adhering to the literal, but not intended, meanings of phrases (8). As many as 65% of autistic children have been reported to have difficulty with reading comprehension (6, 9), which is significantly higher than the 3 to 10% prevalence of comprehension deficits in children that are considered neurotypical (10). With unique needs for social and educational support, many teachers and parents struggle to meet the needs of these children (11, 12). Previous studies have shown that behavioral interventions and reading intervention programs can lead to better language comprehension outcomes for autistic individuals (13, 14). Given the pervasive impact of the language processing differences associated with autism, there is a significant need for designing and testing effective reading intervention programs specific to these needs.

Recent research has established specific profiles of reading abilities in autism (15, 16). For instance, Davidson (16) highlights five distinct reading profiles: no deficits in word reading (i.e., decoding) or comprehension (i.e., *typical readers*), above-average word reading abilities accompanied by below-average comprehension (i.e, *discrepant poor comprehender profile)*, borderline-average to average decoding skills and below-average comprehension (i.e., *below-average poor comprehender profile*), and two *mixed deficit* profiles differentiated by severity. Of particular interest to the current study is the discrepant poor comprehender profile. Difficulties in comprehension may be masked by strong decoding skills and could be unnoticed in younger children until later required to read and comprehend, or make inferences about, longer passages of written material. Thus, such a profile of intact decoding skills accompanied by poor comprehension can have significant impact on academic performance. Additionally, autistic children with higher verbal and intellectual functioning may receive most of their education in the general education classroom (17) and receive school services focusing on behavioral and social skills, which may not meet their academic needs (18).

As comprehension difficulties pose major hurdle in academic performance in many autistic children, it is critical to examine the underlying mechanisms and factors that may contribute to such difficulties. Many cognitive and linguistic processes contribute to reading and text comprehension. The “*Simple View of Reading*” is one theoretical framework that asserts that reading comprehension is determined by word decoding and language comprehension (19, 20). Word decoding is the process of sounding out or recognizing individual words, while language comprehension is the ability to understand and consolidate the definitions of the individual words into a meaningful body of text. Oral language skills (9, 21–24), including oral language comprehension (i.e., receptive grammar; 25), vocabulary (26–28), and morphosyntactic comprehension (28) have been identified as predictors of reading comprehension in autism. However, language delays and impairments are not unique to autism, and there are individual differences in the language development amongst autistic children (29). This theory alone does not fully account for difficulties in reading comprehension for those with intact language and word reading skills. Moreover, decoding and language comprehension are complex phenomena that are influenced by a multitude of factors (30).

The Direct and Indirect Effects Model of Reading (DIER model; 30) expands the Simple View of Reading by considering the effects of several other variables on comprehension, established through extensive research with neurotypical children and those with specific learning disorders. These variables include word reading, listening comprehension, reading fluency, background knowledge, social functioning, higher order cognitive processes (e.g., inferencing, perspective taking, reasoning, and comprehension monitoring), vocabulary, grammatical (i.e., morphosyntactic and syntactic) knowledge, and executive functions (e.g., working memory and attentional control) (31). These variables are presented in a hierarchical model in which vocabulary, morphosyntax, and working memory indirectly support reading comprehension by contributing to word reading abilities. Working memory is also considered a contributing factor to vocabulary and morphosyntax abilities, in turn predicting inferencing, theory of mind, and comprehension monitoring abilities, which lead to listening comprehension skills. Working memory, morphosyntax, and vocabulary are also considered to have smaller direct impacts on listening comprehension. Similarly, Cain, (32) proposes that a multitude of factors influence reading comprehension. Specifically, they provide evidence to support the role of inferencing, comprehension monitoring, memory, and narrative structuring, along with vocabulary, word reading, and verbal IQ in the comprehension of written text. Thus, problems with reading comprehension can arise from various sources. These theoretical frameworks have helped with identifying factors that contribute to reading comprehension in autism.

For example, contextual integration (33), inferencing (34), and self-monitoring (35) have been found to influence reading comprehension in autism. Verbal working memory has been identified to support reading comprehension in neurotypical individuals (36, 37), such that information must be stored and subsequently updated as the text is read. Working memory has also been shown to impact reading comprehension in autism (38), though vocabulary has been suggested as a stronger predictor (28). Traits related to autism – notably social communication differences – have also been linked to reading comprehension (15, 39), above and beyond the role of word recognition and oral language (25). Associated sociocognitive skills such as theory-of-mind (ToM) and inferential thinking, which are usually difficult for autistic children, are argued to influence children’s ability to understand the intentions of the author or characters in the story as well as their ability to learn from their teachers (40–42).

Fewer studies have examined factors that contribute to the abilities observed in the distinct reading profiles mentioned. A tendency to focus on details and consequently exclude the larger context of situations (i.e., Weak Central Coherence Hypothesis) may explain reported strengths in decoding and deficits in reading comprehension in autism (43–45). Autistic children may separate the text out to their most basic details and unintentionally miss the meaning and context of what they are reading. At the neural level, there have been reports of hypoactivation in Broca’s area and hyperactivation in Wernicke’s area and parietal areas in autistic participants during sentence comprehension (46–48), suggesting increased focus on single word level processing rather than integrating them to infer larger meaning, and increased reliance on visuospatial cues. However, some studies suggest that these patterns of local processing in autism are not found consistently (49), which further illustrates the heterogeneity of language processing in autism. Alternatively, the “Complex Information Processing” hypothesis proposes that autistic individuals show impairments in tasks that require a high, but not low, demand for integration of information (50, 51). For example, word decoding requires less integration of information than reading comprehension, thus explaining the deficits in reading comprehension and above-average reading fluency in autism (52). Some have suggested that discrepant autistic readers have no difficulty with implicit inferencing after reading short vignettes (53), and may also have superior sublexical phonological abilities compared to autistic readers without a discrepant reading profile (54). Given the heterogeneity of reading profiles in autism, there is a significant need to continue examining cognitive factors that play a role in the development of distinct reading profiles. This will inform intervention practices, allowing them to become more targeted in their approach such as leveraging individuals’ strengths to bolster areas of difficulty. Further, this can allow families to select interventions that better suit the unique needs of their child.

Although the efficacy of reading interventions is reported to vary, several emerging themes have been identified as the most effective strategies in reading interventions. For example, three key strategies utilized in reading intervention are *anaphoric cueing*, *explicit instruction*, and *student grouping practices* (55). Two additional methods of effective reading intervention used with autistic children are *guided reading* and *providing visual frameworks* (56). Creative methods for reading interventions in autistic children have also been studied in previous literature, such as computer-based interventions, with mixed results (57, 58). The diverse profiles of reading difficulties in autistic children suggest that a combination of different strategies could be the most helpful to meet the needs of individual autistic students. Given the evidence that visuospatial abilities can sometimes be stronger in autistic individuals (48, 59–61), emphasis on using visual aids in intervention practices is a logical approach. Thus, an emerging question is to apply a strength-oriented approach to determine whether a visuospatial reliant intervention could be used for enhancing reading comprehension in autism.

A reading intervention that focuses on building skills in visual imagery to improve comprehension is the *Visualizing and Verbalizing for Language Comprehension and Thinking* (V/V) (62). The V/V program uses gestalt imagery to develop language comprehension, vocabulary, and higher order thinking. This intervention is modeled around the dual coding theory (DCT) of cognition, which asserts that perception consists of a nonverbal system and a verbal system (63, 64). These systems work together by first coding language (the verbal system) and then creating a mental image for objects and events (the non-verbal system). According DCT, reading is separated into the process of understanding written words by the verbal process, and then transforming the written words into a mental image by the nonverbal process, which produces a holistic meaning of the written text. The V/V intervention utilizes the principles of DCT by helping the student produce a gestalt, or concept imagery of the written words. In a sequential manner, the student moves on to understanding sentences, paragraphs, and page imagery. The V/V intervention has been used in previous studies to improve reading comprehension in children with reading disabilities and non-autistic children with poor comprehension skills (65, 66). In addition, several neuroimaging studies from our group have shown improvement in reading comprehension in autistic children following the V/V intervention, which was also accompanied by changes in brain activity and connectivity (67–69). The V/V intervention is a relatively novel area of research that warrants replication and exploration of factors that may influence intervention outcomes. While the above-mentioned studies report neurobiological changes, a comprehensive behavioral and neuropsychological improvement in reading as a result of V/V intervention has not been reported in the literature.

Thus, the primary goal of the current study is to examine the effectiveness, and compare the outcomes, of the V/V intervention in a sample of autistic children (AUT-EXP), a waitlist control group of autistic children (AUT-WLC), all meeting the discrepant poor comprehender reading profile, and an age-and-IQ-matched non-autistic comparison group (non-AUT). Given previous evidence for the role of vocabulary, memory, and social functioning on reading comprehension, we also examined these variables as predictors of reading comprehension, and further analyzed whether group (AUT-EXP vs. AUT-WLC) moderated the effects these variables have on reading comprehension at Time 2. Based on previous empirical evidence and theoretical support, we hypothesized that: 1) there would be differences in cognitive functioning between the autistic and non-autistic groups; 2) when controlling for baseline skills, the AUT-EXP group would have higher reading comprehension scores at Time 2 compared to time 1; and lastly, 3) group would moderate the effects of vocabulary, social skill, and verbal and visual memory on reading comprehension.

## 2 Method

### 2.1 Participants

Thirty-nine autistic children (FSIQ > 70) and a control group of 26 non-autistic (non-AUT) children – matched on age, FSIQ, and performance IQ (PIQ) – participated in this study. Autistic children were randomly selected to be in either the experimental (AUT-EXP) or waitlist control group (AUT-WLC), yielding 22 participants in the AUT-EXP group and 17 participants in the AUT-WLC group. Participant average ages were 10.29 (*SD*=1.54), 11.05 (*SD*=1.15), and 10.38 (*SD*=0.64) for the AUT-EXP, AUT-WLC, and non-AUT groups, respectively. The majority of the sample identified as male (80%). Most participants identified as Caucasian (52.3%), while 26.2% identified as Black, 12.3% identified as Asian, 7.7% identified as multiracial, and 1.5% specified another racial identity. Autistic participants were recruited through several sources throughout Alabama and through the Lindamood-Bell Learning Processes (LBLP) centers across the United States.

One-way ANOVAs were conducted to compare continuous demographic variables (e.g., age) and baseline measures in the AUT-EXP, the AUT-WLC, and non-AUT groups (**Table 1**). The three groups did not significantly differ in age [*F*(2,62)=1.48, *p*=.24], FSIQ [*F*(2,61)=.57, *p*=.57], verbal IQ [*F*(2,61)=2.24, *p*=.12], or PIQ [*F*(2,59)=.59, *p*=.56]. The AUT-EXP and AUT-WLC groups also did not significantly differ in VIQ.

**Table 1:** Assessment Measures.

	Assessment	Source	Measures
**Parent Report nt-Report**	Social Responsiveness Scale (SRS)	(70)	Social functioning
	Social Communication Questionnaire (SCQ)	(71)	Communication skills and social functioning
Eligibility Screening	Weschler Abbreviated Scale of Intelligence (WASI)	(72)	General intelligence (IQ)
	Slosson Oral Reading Test (SORT-R3)	(73)	Word decoding abilities
	Gray Oral Reading Test (GORT-4)	(74)	Reading comprehension
Additional Cognitive Measures	Peabody Picture Vocabulary Test (PPVT-4)	(75)	Receptive vocabulary
	Expressive Vocabulary Test (EVT-2)	(76)	Expressive vocabulary
	Detroit Test of Learning Aptitude-Oral Directions (DTLA-2)	(77)	Oral language comprehension
	Detroit Test of Learning Aptitude-Word Opposites (DTLA-4)	(78)	Word association knowledge
	Symbol Imagery Test (SIT)	(79)	Phonological processing
	Woodcock Reading Mastery Tests – Revised (WRMT-R) Word Attack	(80)	Phonological processing
	Wide Range Assessment of Memory & Learning (WRAML-2)	(81)	Visual and verbal memory

### 2.2 Procedures

All participants were required to meet the inclusion criteria: a Full Scale and Verbal IQ = 70, as measured by the Wechsler Abbreviated Scale of Intelligence (WASI), between 8-13 years of age, and being a native English speaker. The study consisted of pre and post assessment sessions (10-weeks apart), with the V/V intervention in-between. If the child met initial screening criteria and was previously diagnosed with autism by a professional, the child was randomly assigned to the experimental or waitlist-control group at the end of the phone screening. These evaluation reports were thoroughly reviewed, demographic information was obtained, and the initial assessment was scheduled. Assessments lasted between 2.5 and 3 hours and took place at The University of Alabama at Birmingham (UAB). Families who lived outside of Alabama travelled to Birmingham, AL for the two assessments, but received the V/V intervention at the LBLP center closest to their home.

During the first assessment, written informed consent was obtained from the legal guardians of all participants, and written informed assent was obtained from all participants. A set of screening assessments was administered to all participants by an experienced graduate-level clinician or a licensed clinical psychologist. The screening assessments included measures of cognitive functioning [WASI and/or Peabody Picture Vocabulary Test, Fourth Edition (PPVT-4)], autism symptoms [Autism Diagnostic Observation Scale-Second Edition (ADOS-2) and/or Autism Diagnostic Interview-Revised (ADI-R)], and language ability, especially reading ability [Slosson Oral Reading Test - Revised (SORT-R3)] and reading comprehension [Gray Oral Reading Test - 4 (GORT-4)].

### 2.3 Inclusion criteria

Autistic participants were required to meet the following additional inclusion criteria: obtain a score above the 37^th^ percentile on the SORT-R3 and/or a GORT-4 Accuracy score at least in the 25^th^ percentile, receive a GORT-4 Comprehension score below the 37^th^ percentile, and have a Verbal IQ of at least 70. Additionally, autistic participants either had a former diagnosis from a licensed clinical psychologist, accompanied by an ADOS-2 score that fell within the range for autism that was reported by trained, research-reliable personnel, or they scored within the autism range on the ADOS-2 and/or the ADI-R that was administered by a licensed clinical psychologist. The inclusion criteria for non-AUT participants were as follows: aged 8-13 years, no diagnosis of autism or a language disorder, had a decoding and reading comprehension score that was at least in the 37^th^ percentile on the SORT-R3 and GORT-4, respectively, and a Verbal IQ score of at least 70 as measured by the WASI.

### 2.4 Assessment measures

If participants met the inclusion criteria following the screening assessments, they continued with the next set of cognitive measures during the same testing session. These measures included the Expressive Vocabulary Test, Second Edition (EVT-2), Detroit Tests of Learning Aptitude (DTLA-2) Oral Directions and DTLA-4 Word Opposites subtests, Symbol Imagery Test (SIT), Woodcock Reading Mastery Test – Revised (WRMT-R) Word Attack subtest, and the Wide Range Assessment of Memory and Learning (WRAML-2) Visual and Verbal Index subtests (**Table 2**). During the testing session, parents were asked to complete the Social Responsiveness Scale (SRS-2) (70) and Social Communication Questionnaire (SCQ) (71), which measure autism symptom severity and social functioning. Participants in the AUT-EXP group received the V/V intervention after their first testing session, while participants in the AUT-WLC group received the intervention after their second testing session (i.e., when data collection was complete). Alternate forms of the GORT-4, PPVT-4, EVT-2, and WRMT-R were given at the second testing session. Participants in the NT control group received the same assessment battery but were only tested at one time point and did not receive the intervention or any type of reading instruction in between testing.

**Table 2:** Participant Demographics and Group Characteristics.

Group	AUT-EXP *n*=22		AUT-WLC *n*=17		non-AUT *n*=28			
	*M*	*SD*	*M*	*SD*	*M*	*SD*	*F*	*p*
Age	10.29	1.54	11.05	1.15	10.38	0.64		
Full-Scale IQ (FSIQ)	93.0	13.3	95.13	14.89	97.46	11.85	.57	.57
Verbal IQ (VIQ)	90.29	10.6	89.44	12.25	98.07	14.34	2.24	.12
Performance IQ (PIQ)	100.1	19.5	102.56	16.67	97.64	11.77	.59	.56
SRS-2 T Score	77.41	12.09	77.63	12.14	51.00	16.53	26.64	<.001
SCQ Total Score	19.73	8.21	20.75	5.68	5.82	4.76	28.78	<.001

### 2.5 V/V intervention program

The V/V intervention program, designed by Dr. Nanci Bell and developed by the Lindamood-Bell Learning Processes (LBLP), is a language remediation program that has been widely used among children with reading disorders (62). The V/V intervention is built on the principles of Dual Coding Theory (DCT) of cognition, an established scientific theory that postulates that both visual representation and verbal information are necessary for optimal language comprehension (64). Specifically, the V/V method teaches children to form gestalt, or concept images, as they process verbal and written language, with the goal to develop oral and written language comprehension, bolster vocabulary, and foster higher order thinking skills (62). Children are encouraged to visualize the meaning of what they hear and/or read and then verbalize what they are visualizing. This is done in a sequential manner, such that the student progresses from word imagery to sentence, paragraph, and page imagery.

Therapists first show students pictures and gradually introduce various descriptive “structure words” (i.e., shape, size, orientation, perspective), which provide a framework for the students to thoroughly describe the pictures. Once they are proficient at describing the pictures, they progress to visualizing familiar objects and are encouraged to describe them using the structure words. The student later advances to nouns and fantasy images. The next stage entails visualizing single sentences that are verbalized by the therapist. Similarly, this begins with single nouns and advances to imaging the same noun in new situations via simple sentences. The next stage involves sentence-by-sentence imagery. The student visualizes and verbalizes each sentence and the clinician asks choice questions (e.g. ‘Is the [object] big or little?’), which aim at the main (i.e., gestalt) concept of the paragraph. This method is applied to each sentence of a paragraph and the student places a colored square after each sentence, serving as a visual cue for the image that they visualized. At the conclusion of the paragraph, the student provides an image summary by verbally describing each image that was created for each square. Lastly, the student gives a verbal paraphrase (i.e., word summary) of the paragraph. In the following stage, higher order thinking skills are included. The student forms sentence imagery in the same method as the previous stage, while the therapist introduces inferential and main-idea questions, which require the student to make conclusions and think critically about the text. The intervention continues to progress to multi-sentence imaging, followed by paragraph, and whole-page images. For more information, please refer to Bell (62).

Additionally, the V/V teaching approach is guided and is described as “responding to the response” rather than immediately correcting the child when a mistake is made or telling the child they are right or wrong. Children who received the intervention as part of this study received a total of 200-hours of face-to-face instruction, taking place in 4-hour sessions, 5 days per week over the span of 10 weeks. The intervention was free of cost to participants. The intervention was given in a one-on-one, distraction-free environment. A new interventionist administered treatment every hour, and supervisors were present to provide feedback to the interventionists.

### 2.6 Data analysis

Hypothesis 1 was tested using independent two-sample t-tests to examine the differences in scores on cognitive assessments between the non-AUT group and the autistic group (combined AUT-EXP and AUT-WLC) at Time 1. Hypothesis 2 was tested using a repeated measures ANOVA with Time as a within-subject factor and Group (AUT-EXP, AUT-WLC) as the between-subject factor. Hypothesis 3 was tested by computing Pearson correlation coefficients to examine relationships between baseline cognitive assessment scores and Time 2 reading comprehension, along with multiple regression analyses to determine whether group (EXP vs. WLC) moderated the effects of vocabulary (i.e., EVT-2 and PPVT-4 scores), social skills (i.e., SRS-2 and SCQ scores), and memory (i.e., the WRAML-2 visual and verbal memory scores) on reading comprehension at Time 2.

Statistical analyses were performed using SPSS Version 28.0 (IBM, Armonk, NY). Statistical significance was assessed with an alpha threshold of 0.05 unless otherwise noted. Excluding the SRS-2 and SCQ, all assessment scores were converted to standard scores with a mean of 100 and a standard deviation of 15 (**Table 1**). Descriptive statistics were calculated for variables of interest. These included means and standard deviations, medians and interquartile ranges, and counts and percentages, when appropriate. There were no cases with standardized residuals greater than ±3 standard deviations and, therefore, no cases were omitted as outliers. A total of 2.56% of data was missing. Those participants with missing data for specific cognitive assessments were excluded by an analysis-by-analysis basis. A significance threshold of.0083 was used for regression analyses after bonferroni correction to account for multiple comparisons.

## 3 Results

### 3.1 Group characteristics

Two one-way ANOVAs were conducted with SCQ and SRS-2 scores as dependent variables and group (AUT-EXP, AUT-WLC, non-AUT) as the independent variable. There was a significant main effect of group on baseline SCQ [*F*(2,54)=28.78, *p*<.001] and SRS-2 scores [*F*(2,56)=26.64, *p*<.001]. *Post-hoc* analyses indicated that the AUT-EXP and AUT-WLC groups had significantly higher SCQ scores than the non-AUT group, indicating poorer social functioning. These comparisons were both significant at the *p*<.001 level. Similarly, the *post-hoc* comparisons also indicated that the AUT-EXP and AUT-WLC groups had significantly higher SRS-2 scores than the non-AUT group (*p* values <.001), with scores in both autistic groups exceeding the cutoff for clinically significant autistic symptomatology (> 76) (**Table 2**).

### 3.2 Baseline measures

Independent samples t-tests were conducted to compare scores on the following cognitive measures between the autistic group (AUT-EXP and AUT-WLC combined) and the non-AUT control group: PPVT-4, EVT-2, DTLA-2 Oral Directions, DTLA-4 Word Opposites, SIT, WRMT-R Word Attack subtest, WRAML-2 Visual Index and Verbal Index, FSIQ, VIQ, and PIQ. All assessment scores for both groups were normally distributed as assessed by Shapiro-Wilk’s test (all *p* values >.05). At baseline, the AUT group (*M*=78.55, *SD*=17.36) performed poorer than the non-AUT group (*M*=88.93, *SD*=10.92) on the DTLA-2 Oral Directions subtest (*t*(64)=-2.78, *p*=.007). However, Levene’s Test for Equality of Variances revealed that homogeneity of variance was violated (*p*=.007). As such, a non-parametric Mann Witney test was conducted for this comparison, which yielded a significant difference between the groups (U=331, *p*=.008). The two groups significantly differed in VIQ (*t*(63)=-2.57, *p*=.012), and PPVT-4 scores (*t(*64)=-2.58, *p*=.012). Specifically, the AUT group (*M*=89.92, *SD*=11.2) had a lower VIQ than the non-AUT group (*M*=98.07, *SD*=14.34), and showed lower receptive vocabulary on the PPVT (*M*=91.89, *SD*=11.71) than the non-AUT group (*M*=100.96, *SD*=16.86) (See **Table 3** for more details).

**Table 3:** Comparison of Baseline Measures by Diagnostic Group.

Group	AUT		non-AUT		*t*	*p*
	M	SD	M	SD		
FSIQ	93.92	13.85	97.46	11.85	-1.09	.28
VIQ	89.92	11.20	98.07	14.34	-2.57	.012*
PIQ	101.26	18.06	97.64	11.77	.91	.36
SORT-R3	107.08	8.13	109.93	8.94	-1.36	.179
GORT-4	79.87	12.64	101.07	13.08	-6.67	<.001**
PPVT-4	91.89	11.71	100.96	16.86	-2.58	.012*
EVT-2	91.13	12.21	96.79	12.95	-1.82	.073
DTLA-2 Oral Directions	78.55	17.36	88.93	10.92	-2.78^a^ (U=331)	.007^a^ (.008*)
DTLA-4 Word Opposites	92.69	16.54	96.79	15.65	-1.02	.31
Symbol Imagery Test (SIT)	101.34	14.80	98.67	11.23	.79	.43
WRMT-R Word Attack	107.85	11.22	108.75	13.50	-.30	.77

All scores presented are standard scores with a mean of 100 and standard deviation of 15.

a Due to non-normality of variance (p=.007), a non-parametric Mann-Whitney test was conducted.

*Significant differences at the p <.05 threshold.

**Significant differences at the p <.001 threshold.

Significant values are presented in bold font.

Assessment scores were then compared across the three groups: AUT-EXP, AUT-WLC, non-AUT) (see **Table 4** for means). All assessment scores were submitted as dependent variables to a one-way ANOVA with group as the independent variable. The ANOVA revealed a significant main effect of group on Time 1 GORT-4 reading comprehension scores (*F*(2,64)= 24.04, *p*<.001), GORT-4 fluency scores (*F*(2,64)= 3.71, *p*=.03), and GORT-4 accuracy scores (*F*(2,64)=5.63, *p*=.006) (**Table 5**). There was no significant main effect of group on SORT-R3 scores at baseline (*F*(2,64)=1.31, *p*=.28). *Post-hoc* comparisons revealed that the AUT-EXP and AUT-WLC groups had significantly lower GORT-4 comprehension scores than the non-AUT group at baseline (*p* values <.001), consistent with the inclusion criteria for the study which focused recruitment on autistic children with weaker comprehension skills. The AUT-EXP and AUT-WLC groups did not significantly differ in GORT-4 comprehension at Time 1. However, the autistic groups did differ significantly on GORT-4 fluency scores, with the AUT-EXP group showing higher GORT-4 fluency scores (*p*=.05) than the AUT-WLC group. Additionally, the AUT-WLC group had lower GORT-4 accuracy scores than the non-AUT group (*p*=.04), though word reading scores were still in the average range (AUT-WLC GORT-4 Accuracy T1 = 90.88).

**Table 4:** Cognitive Assessment Scores Across Groups.

Group	AUT-EXP *n*=22		AUT-WLC *n*=17		non-AUT *n*=28	
	*M*	*SD*	*M*	*SD*	*M*	*SD*
GORT-4 Comprehension T1	77.05	11.91	83.53	12.96	101.07	13.08
GORT-4 Comprehension T2	86.59	10.51	86.76	16.10	–	–
GORT-4 Fluency T1	102.73	12.79	91.76	16.29	101.96	12.93
GORT-4 Fluency T2	100.91	9.96	96.18	13.98	–	–
GORT-4 Accuracy T1	98.64	8.88	90.88	12.28	101.79	10.82
GORT-4 Accuracy T2	98.64	9.41	97.35	11.74	–	–
SORT-R3 T1	108.14	8.14	105.71	8.15	109.93	8.94
SORT-R3 T2	108.68	7.63	107.06	8.41	–	–
EVT T1	90.41	11.71	92.06	13.12	96.79	12.95
EVT T2	93.68	9.50	94.31	8.88	–	–
PPVT-4 T1	90.68	11.48	93.56	12.18	100.96	16.86
PPVT-4 T2	92.82	10.86	92.24	15.71	–	–
WRAML Verbal Memory	82.71	11.97	83.41	18.50	–	–
WRAML Visual Memory	83.19	18.13	87.94	14.06	–	–
DTLA-2 Word Opposites T1	94.77	14.60	90.00	18.88	96.79	15.65
DTLA-2 Word Opposites T2	96.59	13.13	93.24	16.10	–	–
DTLA-2 Oral Directions T1	78.33	16.91	78.82	18.41	88.93	10.92
DTLA-2 Oral Directions T2	80.45	18.56	87.81	20.73	–	–
WRMT-R Word Attack T1	107.32	8.97	108.53	13.88	108.75	13.50
WRMT-R Word Attack T2	108.36	9.39	109.94	11.81	–	–
Symbol Imagery Test (SIT) T1	100.91	15.54	101.94	14.21	98.67	11.23
Symbol Imagery Test (SIT) T2	104.91	13.91	107.29	15.09	–	–

Word Opposites, Oral Directions, GORT-4 rate, accuracy, fluency and comprehension were converted to standard scores with a mean of 100 and standard deviation of 15. Assessment measures were not administered at a second time point to the non-AUT group.

**Table 5:** Comparison of Cognitive Assessment Scores Across Groups.

Assessment	*df*	*F (t)*	*p*
GORT-4 Comprehension T1	64	24.03	<.001**
AUT-EXP vs. AUT-WLC	37	(-1.62)	.113
AUT-EXP vs. Non-AUT	48	(-6.70)	<.001**
AUT-WLC vs Non-AUT	43	(-4.38)	<.001**
GORT-4 Comprehension T2	37	.002	.97
GORT-4 Fluency T1	64	3.71	.03*
AUT-EXP vs. AUT-WLC	37	(2.36)	.024*
AUT-EXP vs. Non-AUT	48	(.208)	.836
AUT-WLC vs Non-AUT	43	(-2.32)	.025*
GORT-4 Fluency T2	37	1.52	.23
GORT-4 Accuracy T1	64	5.62	.006*
AUT-EXP vs. AUT-WLC	37	(2.29)	.028*
AUT-EXP vs. Non-AUT	48	(-1.10)	.275
AUT-WLC vs Non-AUT	43	(-3.12)	.003*
GORT-4 Accuracy T2	37	.14	.71
SORT-R3 T1	64	1.31	.28
SORT-R3 T2	37	.397	.53
EVT T1	64	1.73	.19
EVT T2	36	.04	.84
PPVT-4 T1	63	3.49	.037*
AUT-EXP vs. AUT-WLC	36	(-.74)	.461
AUT-EXP vs. Non-AUT	48	(-2.45)	.018*
AUT-WLC vs Non-AUT	42	(-1.54)	.132
PPVT-4 T2	37	.019	.89
WRAML Verbal Memory	36	.020	.89
WRAML Visual Memory	36	.78	.38
DTLA-2 Word Opposites	64	.94	.40
DTLA-2 Oral Directions	63	3.8	.027*
AUT-EXP vs. AUT-WLC	36	(-.085) U=176	.932 .941
AUT-EXP vs. Non-AUT	47	(-2.66) U=190.5	.01 .034*
AUT-WLC vs Non-AUT	43	(-2.32) U=140.5	.025 .021*
WRMT-R Word Attack	64	.09	.91
Symbol Imagery Test (SIT)	62	.34	.72

Word Opposites, Oral Directions, GORT-4 rate, accuracy, fluency and comprehension were converted to standard scores with a mean of 100 and standard deviation of 15.

*Significant differences at the p <.05 threshold.

**Significant differences at the p <.001 threshold.

Significant values are presented in bold font.

There was also a significant main effect of group on baseline PPVT-4 scores (*F*(2,63)=3.59, *p*=.037) and DTLA-2 Oral Directions scores (*F*(2,63)=3.81, *p*=.027, **Table 5**). *Post-hoc* analyses yielded significant differences between the AUT-EXP and non-AUT groups only on PPVT-4 scores (*p*=.018). As homogeneity of variance was again violated for DTLA-2 Oral Directions (*p*=.026), non-parametric Mann Witney tests were conducted, which revealed significant differences between the AUT-EXP and non-AUT groups (*p*=.034), as well as the AUT-WLC and non-AUT groups (*p*=.021), consistent with the previous combined group analysis.

### 3.3 Effects of V/V intervention

**Figure 1:**
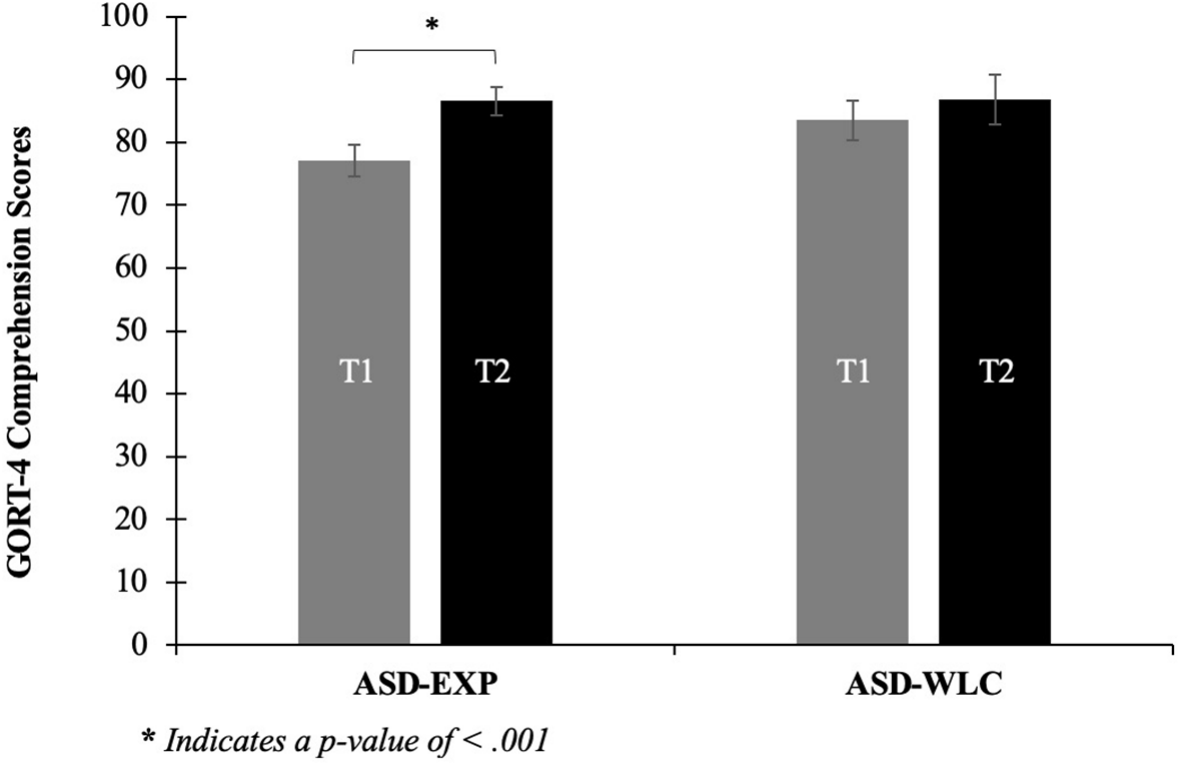
Change in reading comprehension, measured by GORT-4, following V/V intervention. Grey bar: time 1 (pre-test); black bar: time 2 (post-test). Error bars represent standard error of the mean. *Note*. The autistic group that received the intervention in-between testing sessions showed significant improvement in reading comprehension from time 1 to time 2, *p*<.001, *d*=.89.

A repeated measures ANOVA with Time as the within-subject factor and Group as the between subject factor was used to assess the influence of the V/V intervention on children’s reading comprehension (GORT-4 Comprehension scores). Results yielded a group by time interaction that was trending toward significance (F(1,37)=3.08, *p*=.08). The AUT-EXP and AUT-WLC groups did not differ in reading comprehension at Time 1 (*p*=.11) or Time 2 (*p*=.97). However, GORT-4 Comprehension scores significantly differed from Time 1 to Time 2 for the AUT-EXP group (*p*<.001), but not for the AUT-WLC group (*p*=.24) (**Figure 1**).

### 3.4 Group moderating effects

**Figure 2:**
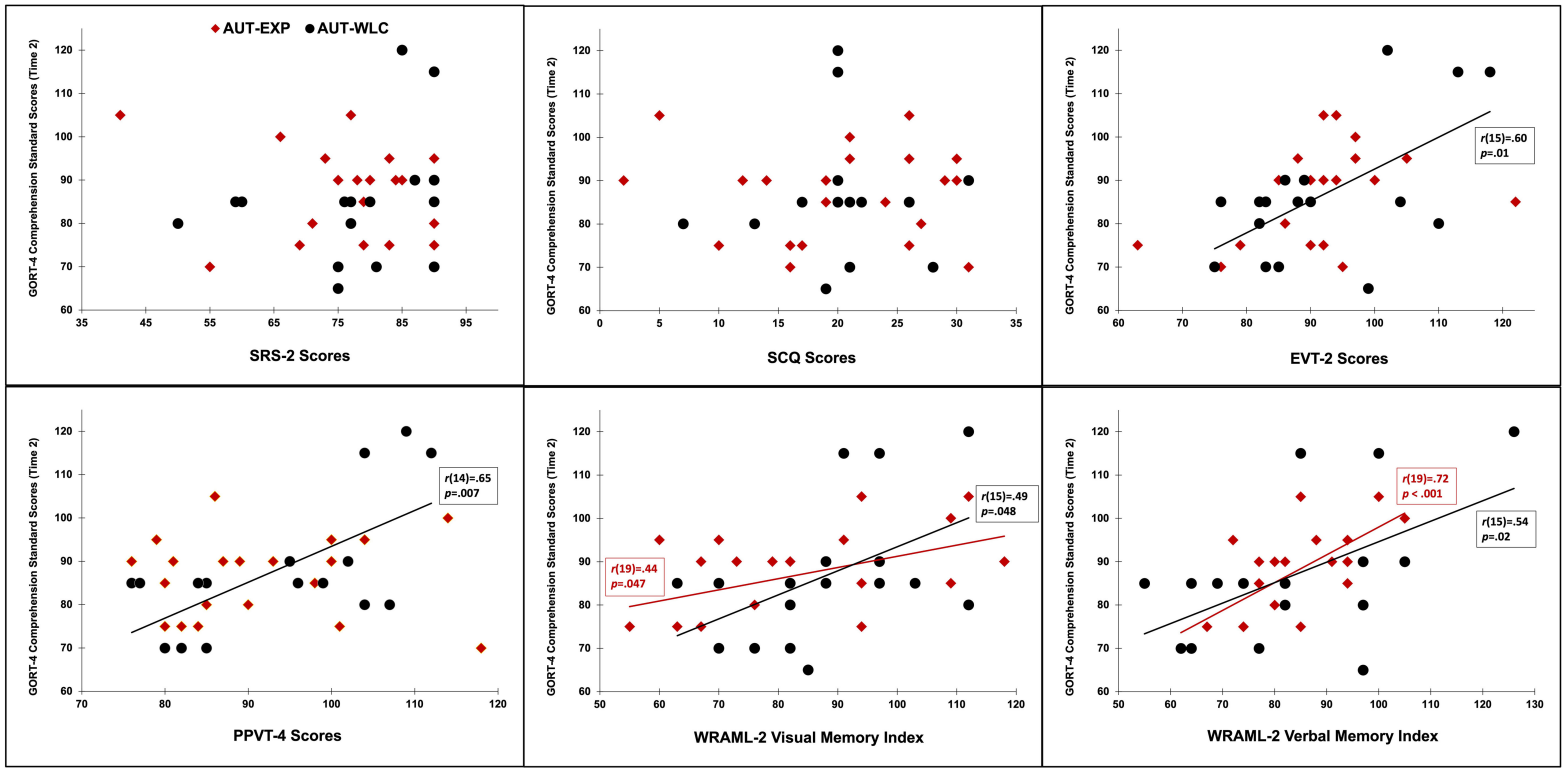
Assessment scores for the AUT-EXP (red) and AUT-WLC (black) groups are depicted. Pearson correlation coefficients were computed to assess relationships between baseline neuropsychological variables and autistic children’s reading comprehension after completing an intervention (AUT-EXP) or not (AUT-WLC). Trendlines depict significant correlations at the *p*<.05 threshold.

Pearson correlation coefficients were computed to assess the relationships between baseline performance on cognitive assessments (i.e., EVT-2, PPVT-4, SRS-2, SCQ, WRAML-2 visual and verbal memory scores) and Time 2 reading comprehension across autistic groups combined, as well as within AUT-EXP and AUT-WLC groups. Across groups, significant, positive correlations were found between Time 2 reading comprehension and baseline PPVT-4 (*r*(36)=.36, *p*=.027), EVT-2 (*r*(37)=.51, *p*<.001), WRAML-2 verbal memory index (*r*(36)=.60, *p*<.001), and visual memory index scores (*r*(36)=.43, *p*=.007). These correlations remained when examined within the AUT-WLC group alone (see **Figure 2**). Within the AUT-EXP group alone, verbal (*r*(19)=.72, *p*<.001) and visual memory (*r*(19)=.44, *p*=.047) were significantly correlated with Time 2 reading comprehension scores.

Multiple regression analyses were used to examine whether group (EXP vs. WLC) moderated the effects of vocabulary (i.e., EVT-2 and PPVT-4 scores), social skills (i.e., SRS-2 and SCQ scores), and memory (i.e., the WRAML-2 visual and verbal memory scores) at Time 1 on GORT-4 comprehension scores at Time 2 (**Figure 2** depicts these scores across groups). A series of regression models were conducted with GORT-4 comprehension scores at Time 2 as the dependent variable. The following variables were entered as simultaneous predictors: SRS, SCQ, PPVT, EVT, WRAML Verbal Memory, and WRAML Visual Memory, and Group (AUT-WLC vs. AUT-EXP). Each regression model differed by the final predictor variable in each model—the interaction term between Group and the focal predictor. None of the interaction terms were significant predictors of GORT-4 comprehension scores at Time 2 (See **Supplementary Table 1**). However, WRAML-2 Verbal Memory scores at time 1 emerged as a significant predictor of GORT-4 comprehension scores at Time 2 in five out of six regression models.

## 4 Discussion

### 4.1 Overview

Our research group has been the first to report the effects of the V/V intervention on reading comprehension in the autism literature. In the current study, we compared differences in cognitive variables between autistic and non-autistic participants, examined the effects of intervention on reading comprehension in autistic children, explored associated predictors, and assessed the group moderating effects. This study found that the AUT-EXP group showed significant improvement in reading comprehension after receiving the intervention, while the AUT-WLC group did not. Both autistic groups had lower baseline reading comprehension compared to the non-AUT group, while only the AUT-EXP group had a lower baseline receptive vocabulary. Significant associations were found between baseline receptive vocabulary, expressive vocabulary, visual memory, and verbal memory with Time 2 reading comprehension across groups, and within the AUT-WLC group, while only verbal and visual memory were significantly correlated within the AUT-EXP group. Lastly, group did not moderate effects of baseline cognitive test performance on reading comprehension at Time 2; although verbal memory emerged as a significant predictor of Time 2 reading comprehension scores across groups.

### 4.2 Reading intervention outcomes

The main goal of the current study was to determine if the V/V intervention can facilitate improvements in reading comprehension in autistic children with poor comprehension. Our group of autistic children improved in reading comprehension after receiving the intervention by.89 standard deviations from Time 1 to Time 2, which is considered a large effect size. This supports previous findings suggesting that the V/V intervention can facilitate reading comprehension improvement in autistic children with average or above-average decoding skills and below-average reading comprehension skills (67–69). Some higher-IQ autistic individuals are skilled in visuospatial processing (59–61) and have been reported to outperform non-autistic peers on tasks, such as the Block Design subtest of the Wechsler Intelligence Scales (82) and/or the Embedded Figures Test (83–85). However, poorer reading comprehension in autistic children may be a result of reduced multisensory integration. Scaffolding children to bridge their enhanced visual processing to verbal systems, supporting principles of the dual coding theory (64), is a strengths-based approach that may be an effective mechanism for facilitating reading comprehension in autism. Examining the direct relationship between performance on visuospatial tasks and improvement in reading comprehension after the V/V intervention may be an avenue for future research.

### 4.3 Predictors of reading comprehension and group moderation

We also tested the hypothesis that group would moderate the effects of cognitive variables on reading comprehension at Time 2. In the current study, none of the interaction terms across regression models were significant, suggesting the intervention did not moderate relationships between cognitive variables and reading comprehension at Time 2. Although previous studies have reported relationships between social functioning and reading comprehension, these effects were not found in the current study. Aspects of social functioning, such as ToM and executive functioning (EF) have been found to account for unique variance in reading comprehension in autism (86). Specifically, shifting contributes to both literal and inferential reading comprehension, whereas ToM contributes uniquely to inferential comprehension only. It is possible that aspects of EF play a greater role in reading comprehension for this specific profile of readers than social functioning, as evidenced by relationships found between verbal memory and comprehension (discussed below). Examining relationships between other aspects of EF, such as cognitive flexibility, and reading comprehension in this population is a recommendation for future research. Future studies should also examine ToM specifically as a predictor of reading comprehension, specifically for questions requiring inferencing, within the discrepant poor comprehender profile to determine the role of perspective-taking on drawing inferences from the text.

When entered simultaneously in regression analyses, baseline verbal memory emerged as a significant predictor of reading comprehension at Time 2 across combined autistic groups, while baseline visual memory skills did not predict reading comprehension at Time 2. This suggests that previous reports of vocabulary being the strongest predictor of reading comprehension in autism (28) may not apply to children within this specific reading profile, as neither expressive nor receptive vocabulary were found as predictors of comprehension above and beyond the other variables. Rather, performance on tasks such as story retelling and word list recall, which require rote short-term storage of verbal information, predicted future reading comprehension scores for our sample. The WRAML verbal memory index scores have been observably lower for children with both current and previous language impairments (87). Of note, our sample of autistic children had a combined average score on this index that was greater than one SD below the population mean (i.e., a standard score of 100). As such, additional supports for verbal information processing may benefit individuals with this specific reading profile, particularly for those with a history of language impairment.

### 4.4 Cognitive variables

When comparing the three groups, the AUT-EXP group had lower baseline vocabulary when compared to non-AUT participants. It should be noted that this pattern was expected given that a below average reading comprehension score was an inclusion criterion for the autistic group. Autistic participants also had poorer performance on the Oral Directions subtest of the DTLA-2 relative to non-AUT participants, but the groups did not differ on any other cognitive variables. In this task, participants must wait to perform a series of oral directions being read by the experimenter until they have been given in entirety, therefore requiring linguistic processing skills and executive functions, such as behavioral inhibition, attention, and memory. However, our participants did not differ on any other cognitive task performance. Marking visual stimuli on paper, a requirement of the oral directions task, involves integration of auditory, visual, and motor processes. Therefore, it is possible that our sample of participants had more difficulty integrating these processes, in line with complex-information processing theory of autism (50).

### 4.5 Limitations and future directions

The current longitudinal study, despite having multiple time points and groups of participants, is limited by a relatively small sample size and multiple comparisons; thus, we suggest that results be interpreted with a degree of caution. In this study, the non-autistic comparison group did not have a discrepant reading profile (i.e., intact word reading skills but poor reading comprehension) and were assessed at only one time point, which permitted only pre-intervention comparisons between these groups. Our future research plans to compare cognitive variables and intervention outcomes in autistic children to non-AUT children who share the discrepant reading profile typically addressed by the V/V intervention in regular practice. Additionally, as our findings suggest associations between verbal memory and reading comprehension, future research should explore the effectiveness of verbal memory skills interventions on reading comprehension gains for this population. Given that the V/V intervention utilizes visualization strategies to support reading comprehension, it would also be interesting to assess visuospatial skills as a moderator of intervention response. Lastly, the effects of this intervention may be compared to other reading interventions to determine if the visualizing aspects of the intervention are specifically influencing comprehension improvement.

## 5 Conclusions

The findings of the current study substantiate the importance of targeted interventions for autistic children and provide support for the use of the V/V intervention to facilitate reading comprehension. As autism is widely heterogeneous in its manifestation, examining social and cognitive variables that influence reading comprehension within specific established profiles is necessary. The current study found that verbal memory is a predictor of reading comprehension for a specific profile of readers that excel at word reading while struggling with comprehension. Interventions that leverage this skill may aid in the development of reading comprehension for autistic children with this reading profile.

## Data availability statement

The original contributions presented in the study are included in the article/**Supplementary Material**. Further inquiries can be directed to the corresponding author.

## Ethics statement

The studies involving humans were approved by University of Alabama at Birmingham Institutional Review Board for Human Use (IRB). The studies were conducted in accordance with the local legislation and institutional requirements. Written informed consent for participation in this study was provided by the participants’ legal guardians/next of kin.

## Author contributions

MB: Conceptualization, Writing – original draft, Writing – review & editing, Formal analysis. CP: Writing – original draft. DM: Writing – review & editing, Project administration. HH: Writing – review & editing. AL: Project administration, Writing – review & editing. AS: Writing – review & editing. SO: Project administration, Supervision, Conceptualization, Writing – review & editing. RK: Conceptualization, Funding acquisition, Methodology, Supervision, Writing – review & editing, Investigation, Project administration.
