# Supplementary material for: Reading comprehension improvement in autism
Source: Front Psychiatry. 2024 Mar 18;15:1292018. doi: 10.3389/fpsyt.2024.1292018 (PMC10982812; doi:10.3389/fpsyt.2024.1292018)
Supplement: Supplementary file 1 [file Table_1.docx]

**Supplementary Material**

| **Supplementary Table 1**  ***Results of Linear Regression Analyses with Simultaneous Entry*** | | | | | | | |
| --- | --- | --- | --- | --- | --- | --- | --- |
| *Model 1* | | | | | | | |
|  |  |  | 95% CI | |  |  |  |
| Variable | Beta | SE | LL | UL | β | *t* | *p* |
| SRS-2 (T-score) | -.42 | .38 | -1.21 | .37 | -.43 | -1.09 | .28 |
| SCQ | .21 | .24 | -.27 | .69 | .13 | .89 | .38 |
| PPVT-4 | -.10 | .16 | -.43 | .24 | .16 | -.61 | .55 |
| EVT-4 | .31 | .16 | -.01 | .63 | .31 | 1.97 | .06 |
| WRAML-2 Verbal Memory | .55 | .13 | .28 | .81 | .69 | 4.26 | <.001^**^ |
| WRAML-2 Visual Memory | -.03 | .11 | -.25 | .20 | -.03 | -.22 | .83 |
| Group | -12.81 | 19.83 | -53.49 | 27.88 | -.53 | -.65 | .52 |
| Group*SRS Interaction | .16 | .25 | -.36 | .68 | .57 | .64 | .53 |
| *Model 2* | | | | | | | |
|  |  |  | 95% CI | |  |  |  |
| Variable | Beta | SE | LL | UL | β | *t* | *p* |
| SRS-2 (T-score) | -.23 | .15 | -.54 | .07 | -.23 | -1.56 | .13 |
| SCQ | -.28 | .66 | -1.65 | 1.08 | -.17 | .43 | .67 |
| PPVT-4 | -.07 | .17 | -.42 | .29 | -.06 | -.38 | .71 |
| EVT-2 | .32 | .15 | .00 | .63 | .32 | 2.05 | .05 |
| WRAML-2 Verbal Memory | .55 | .13 | .29 | .81 | .70 | 4.36 | <.001^**^ |
| WRAML-2 Visual Memory | -.02 | .11 | -.24 | .20 | -.03 | -.18 | .86 |
| Group | -8.74 | 10.89 | -31.09 | 13.60 | -.36 | -.80 | .43 |
| Group*SCQ Interaction | .41 | .51 | -.63 | 1.45 | .50 | .81 | .43 |
| *Model 3* | | | | | | | |
|  |  |  | 95% CI | |  |  |  |
| Variable | Beta | SE | LL | UL | β | *t* | *p* |
| SRS-2 (T-score) | -.19 | .14 | -.48 | .10 | -.19 | -1.33 | .20 |
| SCQ | -.21 | .24 | -.28 | .70 | .13 | .90 | .38 |
| PPVT-4 | -.11 | .17 | -.45 | .24 | -.10 | -.63 | .54 |
| EVT-2 | .32 | .16 | -.003 | .64 | .32 | 2.03 | .05 |
| WRAML-2 Verbal Memory | .62 | .37 | -.14 | 1.37 | .78 | 1.67 | .11 |
| WRAML-2 Visual Memory | -.01 | .11 | -.24 | .21 | -.018 | -.12 | .91 |
| Group | -3.08 | 17.75 | -33.34 | 39.49 | .13 | .17 | .86 |
| Group*Verbal Memory Interaction | -.40 | .21 | -.48 | .39 | -.16 | -.19 | .85 |
| *Model 4* | | | | | | | |
|  |  |  | 95% CI | |  |  |  |
| Variable | Beta | SE | LL | UL | β | *t* | *p* |
| SRS-2 (T-score) | -.19 | .15 | -.50 | .11 | -.19 | -1.29 | .21 |
| SCQ | -.22 | .24 | -.28 | .71 | .13 | .91 | .37 |
| PPVT-4 | -.12 | .16 | -.45 | .22 | -.11 | -.71 | .48 |
| EVT-2 | .32 | .16 | -.002 | .65 | .32 | 2.04 | .05 |
| WRAML-2 Verbal Memory | .55 | .13 | .29 | .82 | .69 | 4.25 | <.001^**^ |
| WRAML-2 Visual Memory | -.009 | .28 | -.59 | .57 | -.01 | -.03 | .97 |
| Group | -.19 | 18.38 | -37.92 | 37.53 | -.01 | -.01 | .99 |
| Group*Visual Memory Interaction | -.001 | .21 | -.43 | .43 | -.004 | -.004 | .99 |
| *Model 5* | | | | | | | |
|  |  |  | 95% CI | |  |  |  |
| Variable | Beta | SE | LL | UL | β | *t* | *p* |
| SRS-2 (T-score) | -.23 | .15 | -.54 | .06 | -.24 | -1.62 | .21 |
| SCQ | -.30 | .25 | .20 | .81 | .18 | 1.23 | .37 |
| PPVT-4 | -.14 | .16 | -..47 | .19 | -.14 | -.86 | .48 |
| EVT-2 | -.05 | .39 | -.84 | .74 | -.05 | -.13 | .05 |
| WRAML-2 Verbal Memory | .55 | .13 | .29 | .81 | .69 | 4.37 | <.001^**^ |
| WRAML-2 Visual Memory | -.03 | .11 | -.25 | .19 | -.04 | -.27 | .97 |
| Group | -24.8 | 23.68 | -73.43 | 23.76 | -1.02 | -1.05 | .99 |
| Group*EVT-2 Interaction | .27 | .26 | -.26 | .81 | 1.09 | 1.05 | .99 |
| *Model 6* | | | | | | | |
|  |  |  | 95% CI | |  |  |  |
| Variable | Beta | SE | LL | UL | β | *t* | *p* |
| SRS-2 (T-score) | -.21 | .14 | -.50 | .09 | -.21 | -1.46 | .16 |
| SCQ | .29 | .26 | -.23 | .81 | .17 | 1.12 | .27 |
| PPVT-4 | -.39 | .43 | -1.28 | .50 | -.38 | -.91 | .37 |
| EVT-2 | .31 | .16 | -.01 | .63 | .31 | 2.02 | .05 |
| WRAML-2 Verbal Memory | .52 | .13 | .25 | .80 | .66 | 3.88 | <.001^**^ |
| WRAML-2 Visual Memory | -.01 | .11 | -.23 | .21 | -.01 | -.095 | .93 |
| Group | -19.20 | 27.63 | -75.88 | 37.50 | -.79 | -.69 | .49 |
| Group*PPVT-4 Interaction | .20 | .30 | -.40 | .81 | .85 | .69 | .50 |
| *Note.* Alpha thresholds for regression analyses were set to .0083 to account for multiple comparisons (6 regression models).  *^**^p<.001, ^*^p<.05.* | | | | | | | |
